# Supplementary material for: Interferon-independent STING signaling promotes resistance to HSV-1 in vivo
Source: Nat Commun. 2020 Jul 7;11:3382. doi: 10.1038/s41467-020-17156-x (PMC7341812; doi:10.1038/s41467-020-17156-x)
Supplement: Supplementary file 1 — Reporting Summary [file 41467_2020_17156_MOESM1_ESM.pdf]

nature research

Corresponding author(s): Russell E. Vance

Last updated by author(s): May 20, 2020

Reporting Summary

Nature Research wishes to improve the reproducibility of the work that we publish. This form provides structure for consistency and transparency in reporting. For further information on Nature Research policies, see our Editorial Policies and the Editorial Policy Checklist.

Statistics

For all statistical analyses, confirm that the following items are present in the figure legend, table legend, main text, or Methods section.

n/a	Confirmed	
☐	☒	The exact sample size (n) for each experimental group/condition, given as a discrete number and unit of measurement
☐	☒	A statement on whether measurements were taken from distinct samples or whether the same sample was measured repeatedly
☐	☒	The statistical test(s) used AND whether they are one- or two-sided<br>*Only common tests should be described solely by name; describe more complex techniques in the Methods section.*
☒	☐	A description of all covariates tested
☒	☐	A description of any assumptions or corrections, such as tests of normality and adjustment for multiple comparisons
☐	☒	A full description of the statistical parameters including central tendency (e.g. means) or other basic estimates (e.g. regression coefficient) AND variation (e.g. standard deviation) or associated estimates of uncertainty (e.g. confidence intervals)
☐	☒	For null hypothesis testing, the test statistic (e.g. F, t, r) with confidence intervals, effect sizes, degrees of freedom and P value noted<br>*Give P values as exact values whenever suitable.*
☒	☐	For Bayesian analysis, information on the choice of priors and Markov chain Monte Carlo settings
☒	☐	For hierarchical and complex designs, identification of the appropriate level for tests and full reporting of outcomes
☒	☐	Estimates of effect sizes (e.g. Cohen's d, Pearson's r), indicating how they were calculated

Our web collection on statistics for biologists contains articles on many of the points above.

Software and code

Policy information about availability of computer code

Data collection	For colocalization analysis, images were exported to Harmony High-Content Imaging and Analysis Software and automated measurements were performed with the Perkin Elmer Harmony Software v.4.6. as detailed in Methods.
Data analysis	For cytometry analysis FlowJo v.10 was used. Statistical analysis was performed using GraphPad Prism 6.

For manuscripts utilizing custom algorithms or software that are central to the research but not yet described in published literature, software must be made available to editors and reviewers. We strongly encourage code deposition in a community repository (e.g. GitHub). See the Nature Research guidelines for submitting code & software for further information.

Data

Policy information about availability of data

All manuscripts must include a data availability statement. This statement should provide the following information, where applicable:

- Accession codes, unique identifiers, or web links for publicly available datasets
- A list of figures that have associated raw data
- A description of any restrictions on data availability

The authors declare that the data supporting the findings of this study are available within the paper and supplementary information.

Field-specific reporting

Please select the one below that is the best fit for your research. If you are not sure, read the appropriate sections before making your selection.

☒ Life sciences ☐ Behavioural & social sciences ☐ Ecological, evolutionary & environmental sciences

For a reference copy of the document with all sections, see nature.com/documents/nr-reporting-summary-flat.pdf

Life sciences study design

All studies must disclose on these points even when the disclosure is negative.

Sample size	No sample size calculation was performed; mice per group was based on availability of specific genotypes and prior experience as to typical variability. For in vitro experiments, please refer to doi: 10.1016/j.chom.2015.05.004 and doi: 10.1084/jem.20082874. For Mtb experiments, please refer to doi: 10.1038/s41564-019-0578-3. For HSV-1 experiments, pleaser refer to doi: 10.1038/ncomms13348.
Data exclusions	No data were excluded in the analysis.
Replication	All experiments were performed at least twice, each yielding similar results.
Randomization	Organisms were assigned to experimental groups based on genotype. We matched gender and age across groups to control for any differences.
Blinding	Investigators were not blinded during data collection and analysis. Scoring was well distinguishable (movement or no movement).

Reporting for specific materials, systems and methods

We require information from authors about some types of materials, experimental systems and methods used in many studies. Here, indicate whether each material, system or method listed is relevant to your study. If you are not sure if a list item applies to your research, read the appropriate section before selecting a response.

Materials & experimental systems

n/a	Involved in the study	
☐	☒	Antibodies
☒	☐	Eukaryotic cell lines
☒	☐	Palaeontology and archaeology
☐	☒	Animals and other organisms
☒	☐	Human research participants
☒	☐	Clinical data
☒	☐	Dual use research of concern

Methods

n/a	Involved in the study	
☒	☐	ChIP-seq
☐	☒	Flow cytometry
☒	☐	MRI-based neuroimaging

Antibodies

Antibodies used	anti-TBK1 (D1B4) (#3504), anti-phospho-TBK1/NAK (Ser172) (D52C2) (#5483), anti-STING (D2P2F) (#13647), anti-phospho-STING (Ser366) (D7C3S) (#19781), anti-phospho-IRF3 (Ser396) (4D4G) (#4947), anti-LC3B (#2775) all purchased from Cell Signaling Technologies. Anti-IRF3 (EP2419Y) (#ab76409) was from Abcam. Secondary anti-rabbit IgG was conjugated to Alexa Fluor- 680 (Invitrogen).<br>APC CD11b (Biolegend, #101212, clone M1/70 dilution 1:100), FITC anti-rat CD90/mouse CD90.1 (Thy-1.1) (Biolegend, #202503, clone OX-7, dilution 1:100), EAAT2/GLT1 (Novus Biologicals, #NBP1-20136SS, dilution 1:100). Secondary donkey anti-rabbit IgG (H+L) PE (eBioscience, #12-4739-81, dilution 1:100).
Validation	APC CD11b (Biolegend, #101212, clone M1/70 dilution 1:100). Each lot of this antibody is quality control tested by immunofluorescent staining with flow cytometric analysis. Manufacturers show C57BL/6 mouse bone marrow cells stained with CD11b (clone M1/70) APC or rat IgG2b, κ APC isotype control. https://www.biolegend.com/en-us/products/apc-anti-mouse-human-cd11b-antibody-345<br><br>FITC anti-rat CD90/mouse CD90.1 (Thy-1.1) (Biolegend, #202503, clone OX-7, dilution 1:100). Each lot of this antibody is quality control tested by immunofluorescent staining with flow cytometric analysis. Manufacturers show Lou rat thymocytes stained with OX-7 FITC. https://www.biolegend.com/en-us/products/fitc-anti-rat-cd90-mouse-cd90-1-thy-1-1-antibody-2412<br><br>EAAT2/GLT1 (Novus Biologicals, #NBP1-20136SS, dilution 1:100). Validation with western blot analysis of cell lysates from rat and mouse brains. Reacts specifically with GLT-1 in rat and human CNS samples. Application for Western Blot and Flow Cytometry.

From Cell Signaling Technologies (used at dilution 1:1000):

anti-TBK1 (D1B4) (#3504). Validation with western blot analysis of HCT116 cell extracts, untreated (-) or TBK1/NAK knock-out (+), using TBK/NAK antibody #3504. Detects endogenous levels of total TBK1/NAK protein from Human, Mouse, Rat, Monkey. Application for western blot and immunoprecipitation. https://www.cellsignal.com/products/primary-antibodies/tbk1-nak-d1b4-rabbit-mab/3504?site-search-type=Products

anti-phospho-TBK1/NAK (Ser172) (D52C2) (#5483). Validation with western blot analysis of extracts from THP-1 cells differentiated with TPA #4174 (80 nM, overnight) followed by treatment with LPS (1 µg/ml), up to 24h, using Phospho-TBK1/NAK antibody (upper). Rabbit mAb detects endogenous levels of TBK1 only when phosphorylated at Ser172. This antibody may cross-react with phospho-IKKε. Species reactivity: Human, Mouse. Application for Western Blotting, Immunoprecipitation, Immunofluorescence (Immunocytochemistry) and Flow Cytometry. https://www.cellsignal.com/products/primary-antibodies/phospho-tbk1-nak-ser172-d52c2-xp-rabbit-mab/5483?site-search-type=Products

anti-STING (D2P2F) (#13647). Validation with western blot analysis of extracts from 293T cells, mock transfected (-), transfected with a construct expressing human STING protein (hSTING; +), or transfected with a construct expressing mouse STING protein (mSTING; +), using STING (D2P2F) antibody. recognizes endogenous levels of total STING protein. Species Reactivity: Human, Mouse. Application: Western Blotting, Immunoprecipitation, IHC-Leica® Bond™ and Immunohistochemistry (Paraffin).
https://www.cellsignal.com/products/primary-antibodies/sting-d2p2f-rabbit-mab/13647?site-search-type=Products

anti-phospho-STING (Ser366) (D7C3S) (#19781). Validation with western blot analysis of extracts from THP-1 cells differentiated with TPA (80 nM, 16 h) and then untransfected (-) or transfected with poly(dA:dT) (5 µg/mL, 3 h) using phospho-STING antibody. recognizes endogenous levels of STING protein only when phosphorylated at Ser366. Species Reactivity: Human. Application: Western blotting. https://www.cellsignal.com/products/primary-antibodies/phospho-sting-ser366-d7c3s-rabbit-mab/19781?site-search-type=Products

anti-phospho-IRF3 (Ser396) (4D4G) (#4947). Validation with western blot analysis of extracts from HT29 and THP1 cells, control or plpC-transfected (1 hour), using phospho-IRF-3 antibody. detects endogenous levels of IRF-3 when phosphorylated at Ser396. Species Reactivity: Human, Mouse. Application: Western blotting.
https://www.cellsignal.com/products/primary-antibodies/phospho-irf-3-ser396-4d4g-rabbit-mab/4947?site-search-type=Products

anti-LC3B (#2775). Validation with western blot analysis of extracts from HeLa cells, mock transfected or transfected with rat LC3B, and from HT-1080 and A20 cells, untreated or chloroquine-treated (50 µM, overnight), using LC3B Antibody. LC3B detects endogenous levels of total LC3B protein. Cross-reactivity may exist with other LC3 isoforms. Stronger reactivity is observed with the type II form of LC3B. Species Reactivity: Human, Mouse, Rat. Application: Western Blotting, Immunofluorescence (Immunocytochemistry) and Flow Cytometry.
https://www.cellsignal.com/products/primary-antibodies/lc3b-antibody/2775?site-search-type=Products

Anti-IRF3 (EP2419Y) (#ab76409) was from Abcam (dilution 1:1000). Validation with western blot analysis of U937, Hela, MCF7 and Jurkat cell lysates. Application: Western Blotting, Immunoprecipitation, Immunohistochemistry (Paraffin) and Flow Cytometry.
https://www.abcam.com/irf3-antibody-ep2419y-ab76409.html

Animals and other organisms

Policy information about studies involving animals; ARRIVE guidelines recommended for reporting animal research

Laboratory animals	Mus musculus. C57BL/6 (B6), STING gt, STING S365A, STING delta CTT, Tbk1–/–Tnfr1–/–, Irf3–/– , Tnfr1–/– . Males and females, 8-10 weeks old.
Wild animals	No wild animals were used in this study.
Field-collected samples	No field-collected samples were used in this study.
Ethics oversight	All animal experiments complied with the regulatory standards of, and were approved by, the University of California Berkeley Institutional Animal Care and Use Committee.

Note that full information on the approval of the study protocol must also be provided in the manuscript.

Flow Cytometry

Plots

Confirm that:

- [x] The axis labels state the marker and fluorochrome used (e.g. CD4-FITC).
- [x] The axis scales are clearly visible. Include numbers along axes only for bottom left plot of group (a 'group' is an analysis of identical markers).
- [x] All plots are contour plots with outliers or pseudocolor plots.
- [x] A numerical value for number of cells or percentage (with statistics) is provided.

Methodology

Sample preparation	See description in Methods. Briefly, perfused lungs were strained through 40um cell strainers to obtain single cells suspensions.
Instrument	LSR Fortessa X20 (BD)
Software	FlowJo v.10.
Cell population abundance	Microglia represented 3-8%, astrocytes 1-2% and neurons 0.5-2% of total cells post-sorting. Cells were validated through the expression of specific cell markers.
Gating strategy	See gating strategies in Supplementary Information.

- [x] Tick this box to confirm that a figure exemplifying the gating strategy is provided in the Supplementary Information.
