# Supplementary material for: Interferon-independent STING signaling promotes resistance to HSV-1 in vivo
Source: Nat Commun. 2020 Jul 7;11:3382. doi: 10.1038/s41467-020-17156-x (PMC7341812; doi:10.1038/s41467-020-17156-x)
Supplement: Supplementary file 2 — Supplementary Information [file 41467_2020_17156_MOESM2_ESM.pdf]

Supplementary information

Interferon-independent STING signaling promotes HSV-1 resistance *in vivo*

Yamashiro et al.

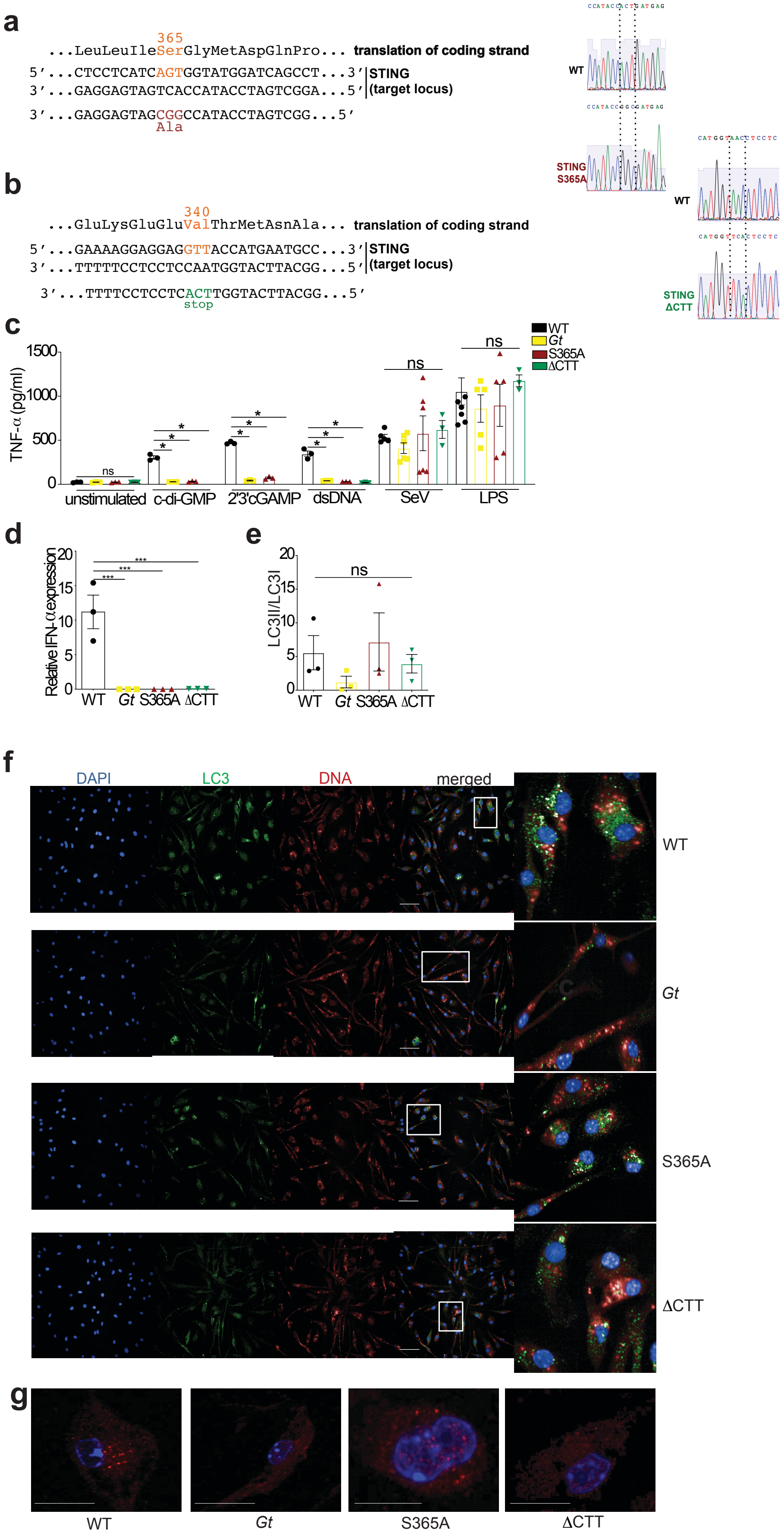
a
365
...LeuLeuIleSerGlyMetAspGlnPro... translation of coding strand
5'...CTCCTCATCAGTGGTATGGATCAGCCT...3' STING
3'...GAGGAGTAGTCACCATACCTAGTCGGA...5' (target locus)
3'...GAGGAGTAGCGGCCATACCTAGTCGG...5'
Ala
WT
STING S365A
b
340
...GluLysGluGluValThrMetAsnAla... translation of coding strand
5'...GAAAAGGAGGAGGTTACCATGAATGCC...3' STING
3'...TTTTTCCTCCTCCAATGGTACTTACGG...5' (target locus)
3'...TTTTCCTCCTCACTTGGTACTTACGG...5'
stop
WT
STING ΔCTT
c
TNF-α (pg/ml)
WT
Gt
S365A
ΔCTT
ns
unstimulated
c-di-GMP
2'3'cGAMP
dsDNA
SeV
LPS
d
Relative IFN-α expression
WT
Gt
S365A
ΔCTT
e
LC3II/LC3I
ns
WT
Gt
S365A
ΔCTT
f
DAPI
LC3
DNA
merged
WT
Gt
S365A
ΔCTT
g
WT
Gt
S365A
ΔCTT

Supplementary Fig. 1. (related to figure 1). **a,** Creation of STING S365A and **b,** ΔCTT mice using CRISPR/Cas9. **c,** Bone marrow derived macrophages (BMM) were stimulated for 6h and TNF-α was measured on the supernatant. **d,** Primary macrophages were transfected with 2´3´cGAMP for 4h and relative expression of *Ifna* was analyzed. **e,** Quantification of LC3II/LC3I ratio from three independent experiments similar to Fig.1d. **f,** Colocalization of DNA and LC3 is increased in WT and S365A cells. Fluorescence images of primary macrophages transfected for 6h with Cy3-labeled DNA and LC3. Images were analyzed by an automated pipeline created on Perkin Elmer Harmony software for colocalization quantification (for more details refer to Methods). Scale bars are 50 µm. **g,** BMM were transfected with 2´3´cGAMP for 4h and STING puncta formation (in red) was visualized. Scale bars are 10 µm. Images were taken using a Carl Zeiss LSM710 confocal microscope. Representative results of three independent experiments, each yielding similar results. Center and error bars show mean and SEM. Analyzed with one-way ANOVA and Tukey post-test. *, $p \leq 0.05$. ns, not significant. Exact p-values were provided in the Supplementary information.

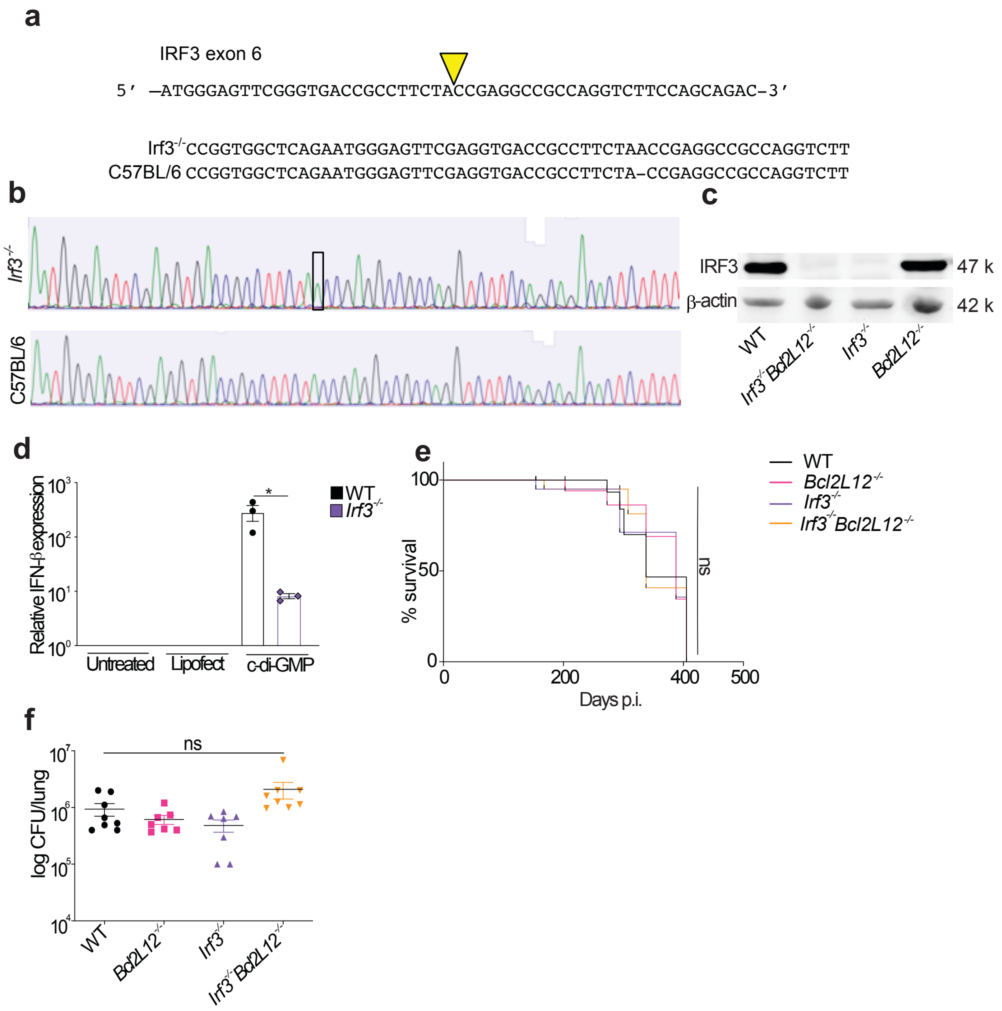

Supplementary Fig. 2. (related to figure 2). Creation of IRF3 deficient mice using CRISPR/Cas9. **a,** CRISPR/Cas9 targeting strategy for IRF3. **b,** Sequencing of the targeted locus resulting in *Irf3*$^{-/-}$ mutation. **c,** Immunoblot of MEFs for IRF3. Representative results of two independent experiments **d,** Primary macrophages were transfected with c-di-GMP for 6h and relative expression of *Ifnb* was analyzed. **e,** Mice were aerosol infected with 400 CFU dose of *M. tuberculosis* (Erdman strain). Survival of infected mice. (n=12 mice per genotype) **f,** Bacterial burden from lungs at 21 days post-infection. n=4 mice per genotype. Combined two independent experiments. All mice except C57BL/6J WT were bred in-house. Four independent experiments were performed, each yielding similar results. Center and error bars show mean and SEM. Analyzed with one-way ANOVA and Tukey post-test. *, $p \leq 0.05$. ns, not significant. Exact p-values were provided in the Supplementary Information.

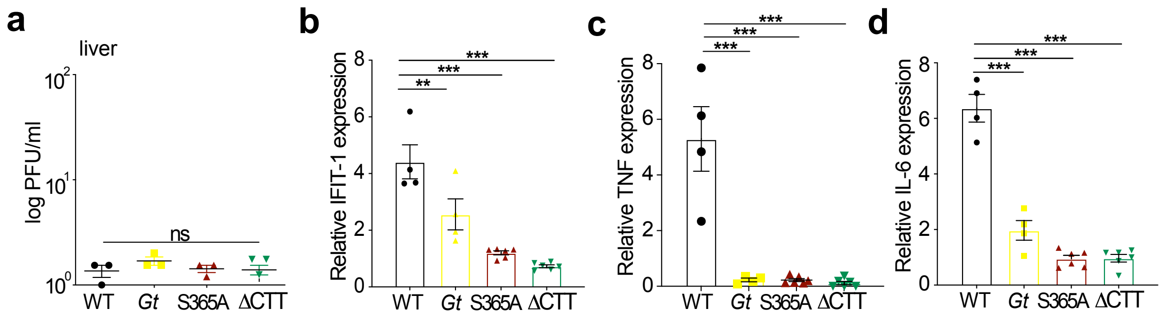

Supplementary Fig. 3. (related to figure 3). Mice were intravenously infected with 1x10^6 PFU of HSV-1 (KOS strain). **a,** Viral titers in the liver at 6 days p.i. **b,** Relative expression of *Ifit1* **c,** *Tnf* and **d,** *Il6* from brains at 3 days p.i. All mice except C57BL/6J WT were bred in-house. **(a)** n=3/3/3/3, **(b-d)** n=4/4/6/6. Representative results of five independent experiments, each yielding similar results. Center and error bars show mean and SEM. Analyzed with one-way ANOVA and Tukey post-test. **, p ≤ 0.005; ***, p ≤ 0.0001. ns, not significant. Exact p-values were provided in the Supplementary Information.

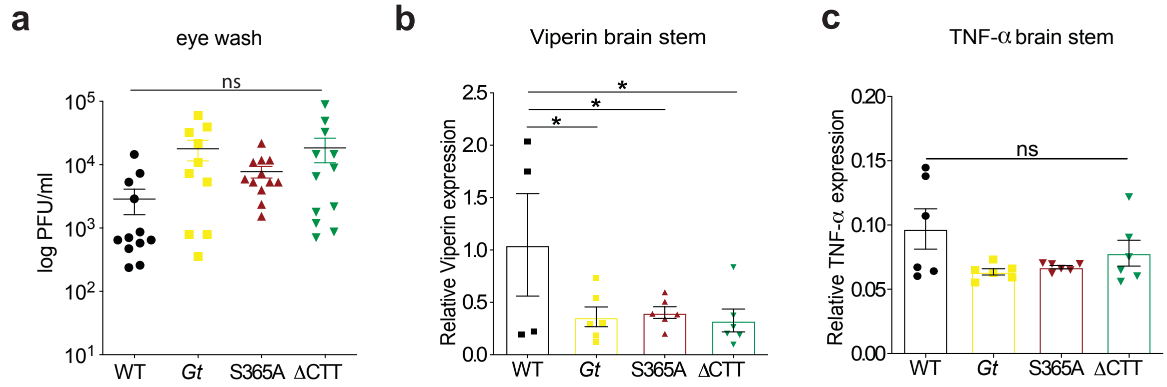

Supplementary Fig. 4. (related to figure 4). Mice were ocular infected with 1x10^5 PFU of HSV-1 (strain 17). **a,**Viral titers from eyes washed at 2 days p.i. Combined three independent experiments, each yielding similar results**. b,** Relative expression of *viperin* and **c,** *Tnfa.* All mice except C57BL/6J WT were bred in-house. **(b)** n=4/6/6/6, **(c)** n=6/6/6/6. Representative results of three independent experiments, each yielding similar results. Center and error bars show mean and SEM. Analyzed with one-way ANOVA and Tukey post-test. *, p ≤ 0.05. ns, not significant. Exact p-values were provided in the Supplementary Information.

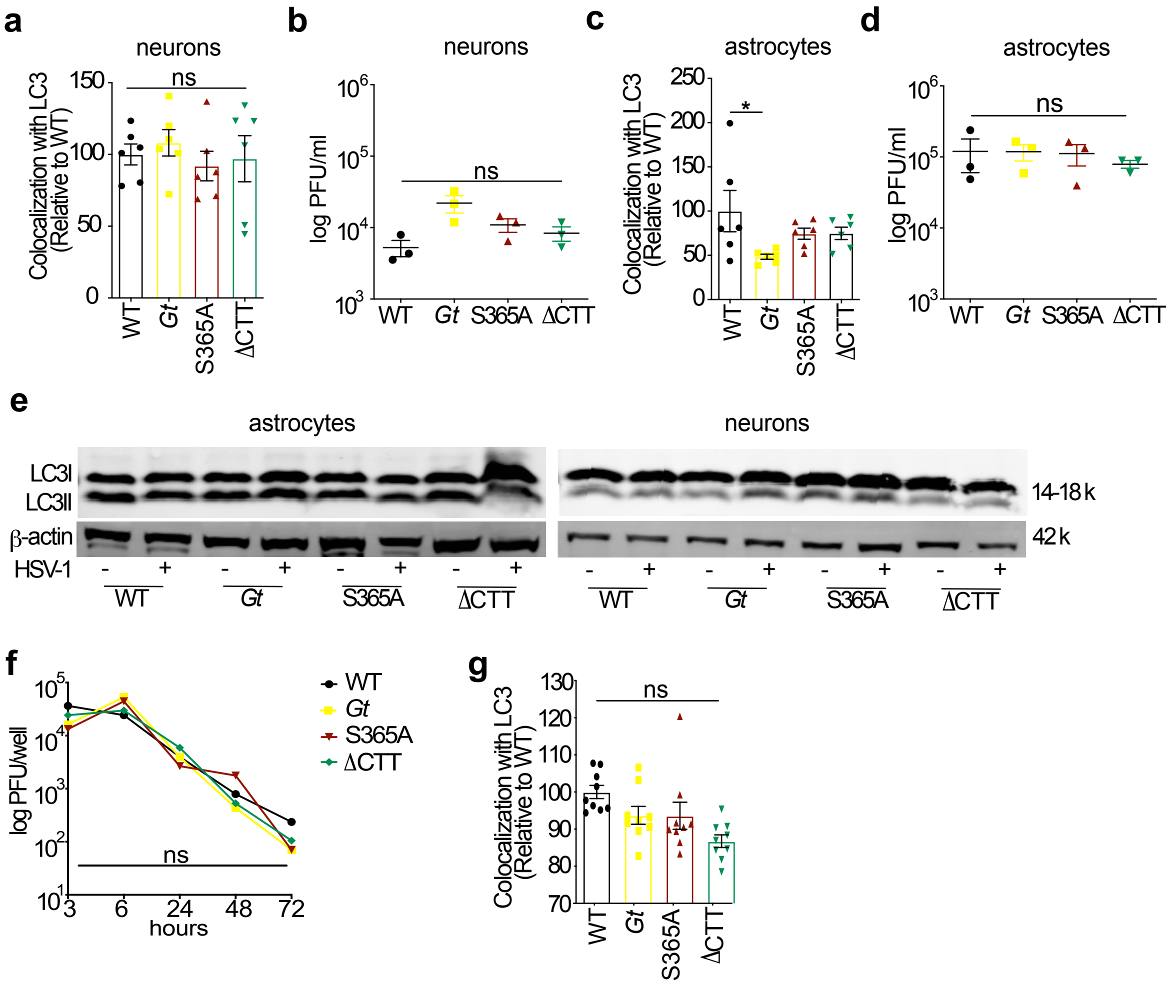

Supplementary Fig. 5. (related to figure 4). Brain cells **(**neurons and astrocytes) were harvested from P0 pups and infected with HSV-1 (KOS strain) at a MOI 1 for 6h and later were stained for LC3 and HSV-1. **a,** Quantification of colocalization of LC3-HSV-1 in neurons was performed and **b,** Viral titers from supernatants were collected 48h later and quantified by TCID50 assay. **c-d,** Same as a-b, in astrocytes. **e,** Cell lysates were collected at 4h post-infection and immunoblot for LC3 and β-actin was performed. **f,** Bone marrow-derived macrophages were infected with HSV-1 (KOS strain) at MOI of 1 and viral titers were quantified at indicated time points. **g,** Quantification of colocalization of LC3-HSV-1 in bone marrow-derived macrophages was performed. **(b and d)** n=3/3/3/3. **(a-g)** Representative results from two independent experiments, each yielding similar results. Center and error bars show mean and SEM. Analyzed with one-way ANOVA and Tukey post-test. *, $p \leq 0.05$. ns, not significant. Exact p-values were provided in the Supplemental Information.

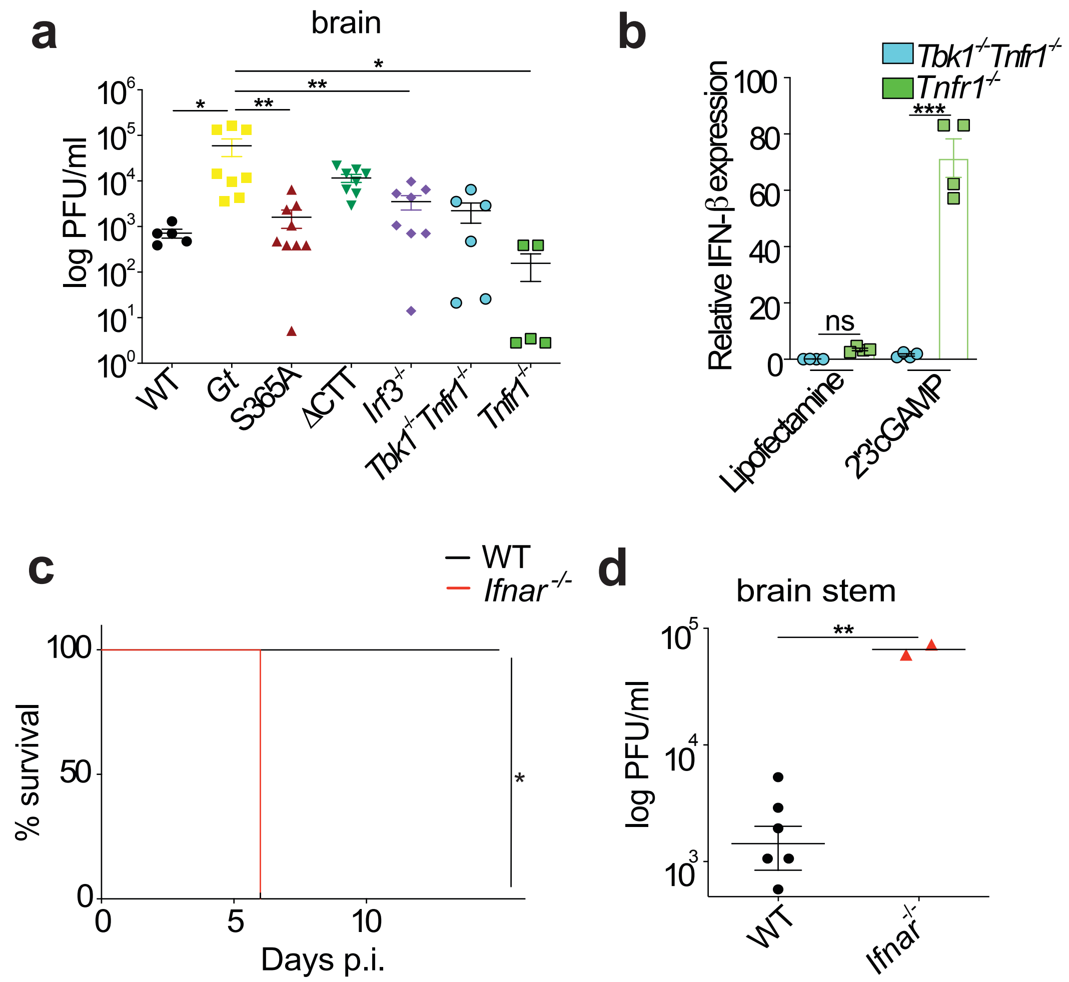

Supplementary Fig. 6. (related to figure 5). **a,** Mice were ocular infected with $1x10^5$ PFU of HSV-1 (strain 17) and viral titers measured in the brain 6 days p.i. **b**, BMDMs were transfected with 2′3′cGAMP for 6h and relative expression of *Ifnb* was analyzed. **c,** Mice were ocular infected with $1x10^5$ PFU of HSV-1 (strain 17) and survival rate and **d,** viral titers measured in the brain stem 6 days p.i. All mice except C57BL/6J WT were bred in-house. **(a)** n=3/5 mice per genotype. Combined results from two independent experiments, each yielding similar results. **(c-d)** n=6/2. Representative results of two independent experiments, each yielding similar results. Center and error bars show mean and SEM. Analyzed with one-way ANOVA and Tukey post-test. *, $p \leq 0.05$; **, $p \leq 0.005$. ns, not significant. Exact p-values were provided in the Supplemental Information.

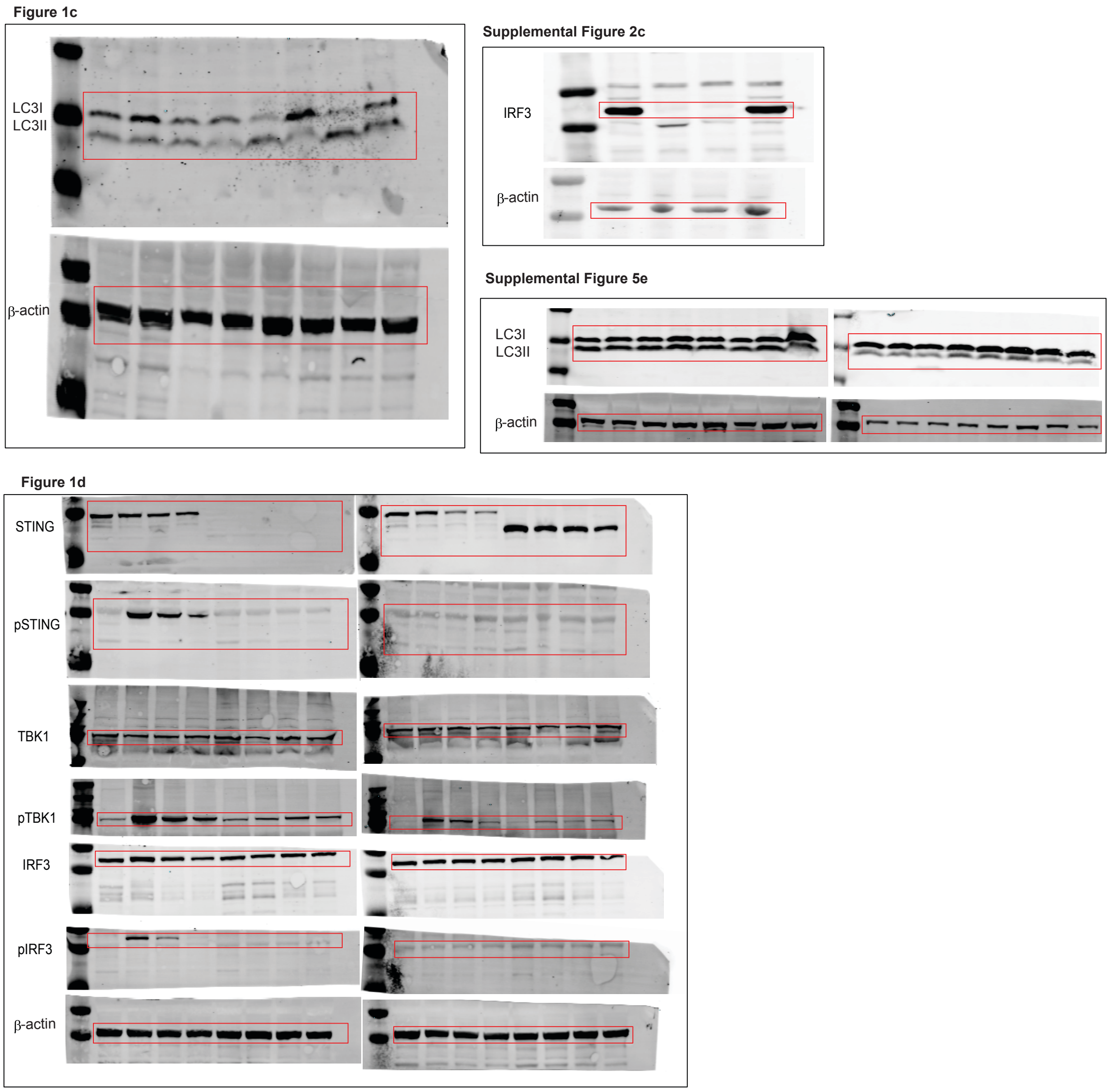

Supplementary Fig 7. Full blots of corresponding figures.

a

Gating strategy for Figure 2g-I (CBA)

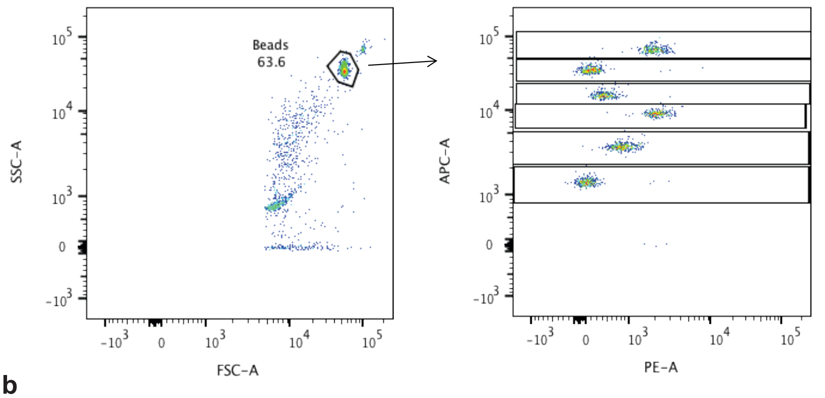

b

Gating strategy for Figure 4g-i (sorted brain cells)

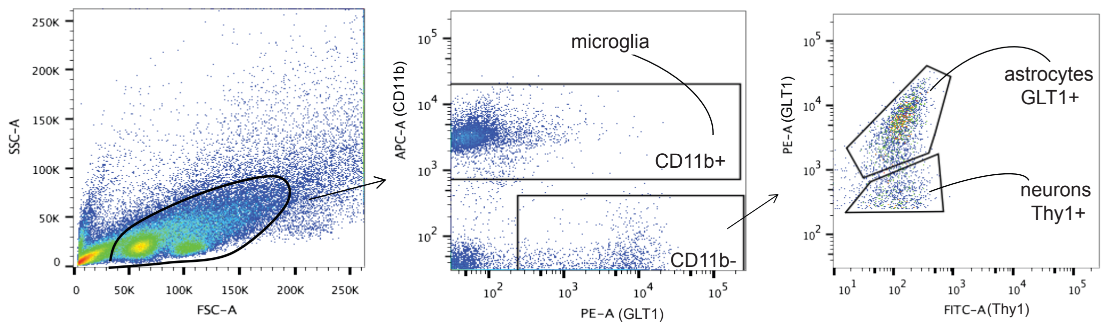

Supplementary Fig 8. Gating strategies. a, Gating strategy for CBA analysis. Beads are selected and analyzed according to APC versus PE staining. Each of the 6 clusters evidenced by APC staining corresponds to one cytokine. Median of intensity of PE staining is plotted to its correspondent standard curve for concentration levels. **b, Gating strategy for sorting of brain cells.** Brains from HSV-1 ocular infected mice were collected on day 3 p.i. and sorted as shown. n=3 mice per genotype. Representative results of three independent experiments, each yielding similar results.

Figure 1			
1a	WT-Gt	WT- S365A	WT-ΔCTT
c-di-GMP	0.01336	0.00848	0.00848
2'3' cGAMP	0.0719	0.0719	0.0719
dsDNA	0.0208	0.0208	0.0208
1b	WT-Gt	WT- S365A	WT-ΔCTT
	0.006	0.0227	0.006
1f	WT-Gt	WT-ΔCTT	
	<0.0001	0.0006	
1g	WT-Gt	WT-ΔCTT	
	<0.0001	<0.0001	
1h	WT-Gt	WT-ΔCTT	
	<0.0001	0.0002	

Suppl 1			
1c	WT-Gt	WT- S365A	WT-ΔCTT
c-di-GMP	<0.0001	<0.0001	<0.0001
2'3' cGAMP	<0.0001	<0.0001	<0.0001
dsDNA	<0.0001	<0.0001	<0.0001
1d	WT-Gt	WT- S365A	WT-ΔCTT
	<0.0001	<0.0001	<0.0001

Suppl 2	
2d	0.0393

Figure 3			
3a	WT-Gt	WT-ΔCTT	
day 5	< 0.01	< 0.001	
day 6	< 0.001	< 0.001	
day 7	< 0.001	< 0.001	
day 8	< 0.001	< 0.001	
3b	WT-Gt	WT-ΔCTT	
day 5	< 0.05	< 0.001	
day 6	< 0.001	< 0.001	
3c	WT-Gt	WT-ΔCTT	
	0.0253	0.0253	
3d	WT-Gt	WT-ΔCTT	
	0.0459	0.0275	
3e	WT-Gt		
	0.0095		
3f	WT-Gt	WT- S365A	WT-ΔCTT
	<0.0001	<0.0001	<0.0001
3g	WT-Gt	WT- S365A	WT-ΔCTT
	<0.0001	<0.0001	<0.0001

Suppl 3			
3b	WT-Gt	WT- S365A	WT-ΔCTT
	0.0054	< 0.0001	< 0.0001
3c	WT-Gt	WT- S365A	WT-ΔCTT
	< 0.0001	< 0.0001	< 0.0001
3d	WT-Gt	WT- S365A	WT-ΔCTT
	<0.0001	<0.0001	<0.0001

Figure 4			
4a	WT-Gt	WT- S365A	WT-ΔCTT
day 6	< 0.001		
day 7		< 0.001	< 0.01
day 8		< 0.001	< 0.01
day 9		< 0.001	
4b	WT-Gt	WT- S365A	WT-ΔCTT
	0.0082	0.0486	0.0016
4c	WT-Gt	WT-ΔCTT	
	0.0011	0.0467	
4d	WT-Gt	WT-ΔCTT	
	< 0.0001	0.048	
4e	WT-Gt	WT-ΔCTT	
	0.0059	0.049	
4f	WT-Gt	WT- S365A	WT-ΔCTT
	0.041	0.046	0.048
4g	WT-Gt	WT- S365A	WT-ΔCTT
	0.0383	0.0133	0.0475
4h	WT-Gt	WT- S365A	WT-ΔCTT
	0.0204	0.0105	0.0094
4i	WT-Gt	WT- S365A	WT-ΔCTT
	0.02768	0.02641	0.02641

Suppl 4			
4b	WT-Gt	WT- S365A	WT-ΔCTT
	0.0335	0.0488	0.027

Figure 5						
5a	WT-Gt	WT-S365A	WT-ΔCTT	WT-Irf3-/-	WT-Tbk1-/-Tnfr1-/-	
day 5	< 0.05					
day 6	< 0.05		< 0.01	< 0.01	< 0.05	
day 7		< 0.01	< 0.001	< 0.001		
day 8		< 0.001		< 0.001		
day 9		< 0.01				
day 11		< 0.01				
day 12		< 0.01				
5b	WT-Gt	WT-ΔCTT	WT-Tbk1-/-Tnfr1-/-			
	0.0027	0.0047	0.0027			
5c	WT-Gt	WT-ΔCTT	WT-Tbk1-/-Tnfr1-/-			
	0.0012	0.049	0.049			
5d	WT-Gt	WT-S365A	WT-ΔCTT	WT-Irf3-/-	WT-Tbk1-/-Tnfr1-/-	WT-Tnfr1-/-
	< 0.0001	< 0.0001	< 0.0001	0.0023	< 0.0001	0.002

Suppl 5	WT-Gt
5c	0.0241

Suppl 6				
6a	WT-Gt	Gt- S365A	Gt-Irf3-/-	Gt-Tnfr1-/-
	0.0115	0.0020	0.0052	0.0106
6b	< 0.0001			
6c	0.0143			
6d	0.0206			

Table 1: P values for corresponding figures.
